# Supplementary material for: Dynamic control of laser driven electron acceleration in a photonic structure using programmable optical pulses
Source: Nat Commun. 2026 Jul 8;17:8424. doi: 10.1038/s41467-026-75233-z (PMC13478337; doi:10.1038/s41467-026-75233-z)
Supplement: Supplementary file 1 [file 41467_2026_75233_MOESM1_ESM.pdf]

Dynamic control of laser driven electron acceleration in a photonic structure using programmable optical pulses

In the supplementary material we highlight the opportunities enabled by the full phase and amplitude control on the laser offered by the use of the LCM in combination with pulse front tilt illumination via particle tracking simulations of the DLA process. In particular, we can simulate the energy gain and particle phase space of electron beams in three different cases. Simulation parameters are listed in Supplementary Table 1. In all cases, the beam is assumed to have uniform density across input phases and the structure has constant periodicity.

First, we look at the electron beam after it passes through a DLA with no phase matching applied. In this case we apply a linear constant phase tilt, achievable in principle by adjusting the angle of incidence without the use of an SLM, in order to roughly tune the resonant energy. In the dark blue plots of Supplementary Fig. 1 we illustrate the input phase and amplitude of this case. The peak accelerating gradient in the 400 nm gap channel is 200 MeV/m; with a total structure length of 5 mm this should result in 1 MeV of energy gain. However, the lack of phase matching as well as strong defocusing forces within the channel instead result in none of the particles being accelerated to that near that energy.

In the simulation shown in cyan in Supplementary Fig. 1, in order to accelerate more of the electron bunch we implement a combination of velocity bunching and

		No Tapering	Ponderomotive, Tapered	APF, Tapered
Electron Parameters	Initial γ	12	12	12
	RMS Spot Size (μm)	1	1	1
	Normalize Emittance (nm-rad)	1	1	1
Structure Parameters	Structure Length (mm)	5	5	5
	Structure Gap (μm)	400	400	1200
	Structure Factor (κ)	0.2	0.2	0.12
	Wavelength (nm)	780	780	780
Laser Parameters	Electric Field (GV/m)	2.4	10	10
	Wavelength (nm)	780	780	780
	Buncher Length (mm)	N/A	0.05	0.1
	Drift Length (mm)	N/A	0.95	0.9

Supplementary Table 1 Simulation Parameters

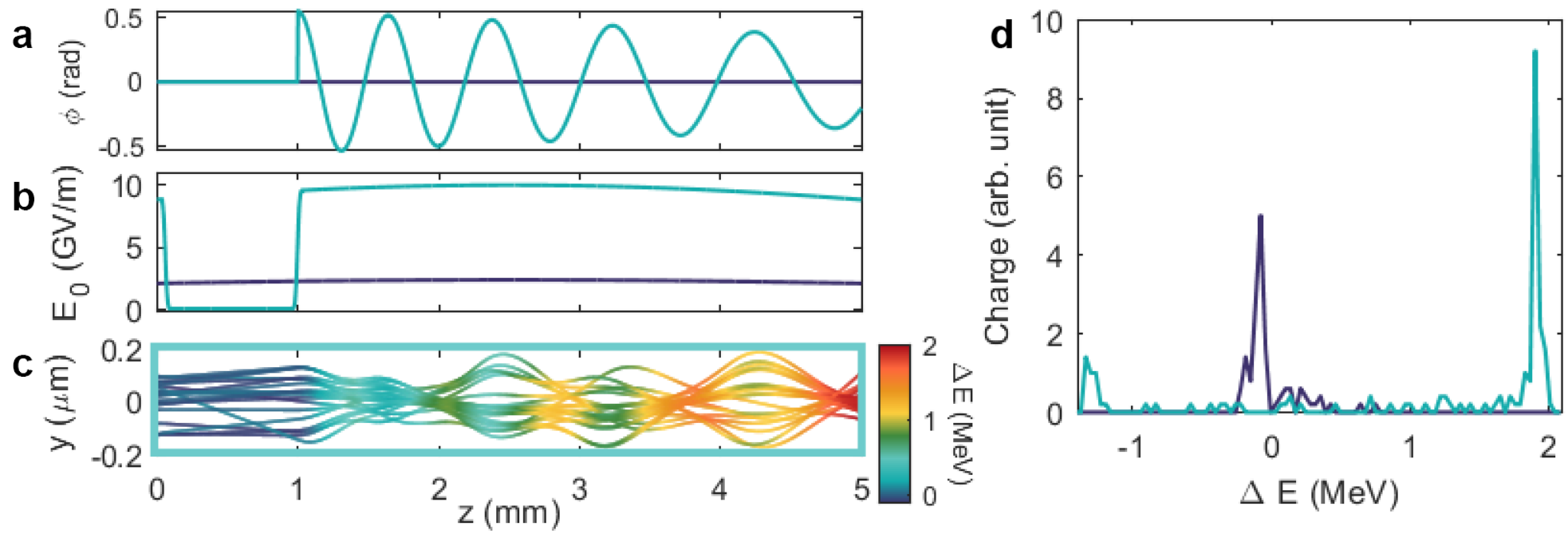

Supplementary Figure 1 **a** Phase and **b** Amplitude inputs for flat (dark blue) and ponderomotive (teal) simulations. **c** Particle trajectories and energy gains are illustrated for the ponderomotive case. **d** Simulated spectra corresponding to the phase and amplitude profiles shown in **a-b**, demonstrating that significantly more particles can be accelerated using a combined bunching and focusing scheme.

ponderomotive focusing by shaping the field amplitude and phases seen by the electrons. Velocity bunching is accomplished by separating the available real estate on the dual grating structure into a prebunching section and a fully matched and tapered accelerating section. The field amplitude is shown Supplementary Fig. 1b. A short initial high-intensity region is used to impart an energy modulation on the beam. This is followed by a low-field region in which the higher energy particles at the rear of the beam 'catch up' to the lower energy particles at the front, converting the energy modulation into a phase-locked density modulation and maximizing the number of particles at the accelerating phase in the downstream section of the structure. In practice, this can be done by using the SLM to apply a properly shaped z-dependent Fresnel lens phase mask $\phi_f(x,z) = -\frac{k_0}{2}\left(\frac{x^2}{f_x(z)}\right)$. At the same time, a ponderomotive focusing scheme is implemented by adding a sinusoidal oscillation on the phase, thus exciting different spatial harmonics which can accelerate and focus the beam respectively, as described by Naranjo et al.[1]. In this simulation the amplitude and period vary between $0.55 \geq A_p \geq 0.35$ and $600\mu\text{m} \leq T_p \leq 860\mu\text{m}$ respectively in order to maximize transverse acceptance while retaining a stable longitudinal bucket, as well as maximize the average gradient throughout the DLA. In Supplementary Fig. 1c we can see that in the initial bunching section the particle energy remains constant as the bunch longitudinally compresses, after which the beam alternately gains and loses energy as it swaps between transversely and longitudinally focusing. Note that both the implementation of longitudinal bunching and ponderomotive focusing come at the expense of energy gain and for this case the incident field is increased to 10 GV/m. The result is that 70% of the transmitted beam is accelerated to the resonant energy, compared to 0% in the flat laser case.

Moving to the APF case, where the $n = 0$ harmonic is used for acceleration, we can use the lattice description found in [2] to optimize the APF period for a given beam energy and laser gradient. For the same electron, structure, and laser parameters of the simulated ponderomotive case, we find even for the optimized APF lattice, with $\phi_{APF} = \pi/3$ the beam envelope for a $\gamma = 12$ beam is near 390 nm, and it becomes

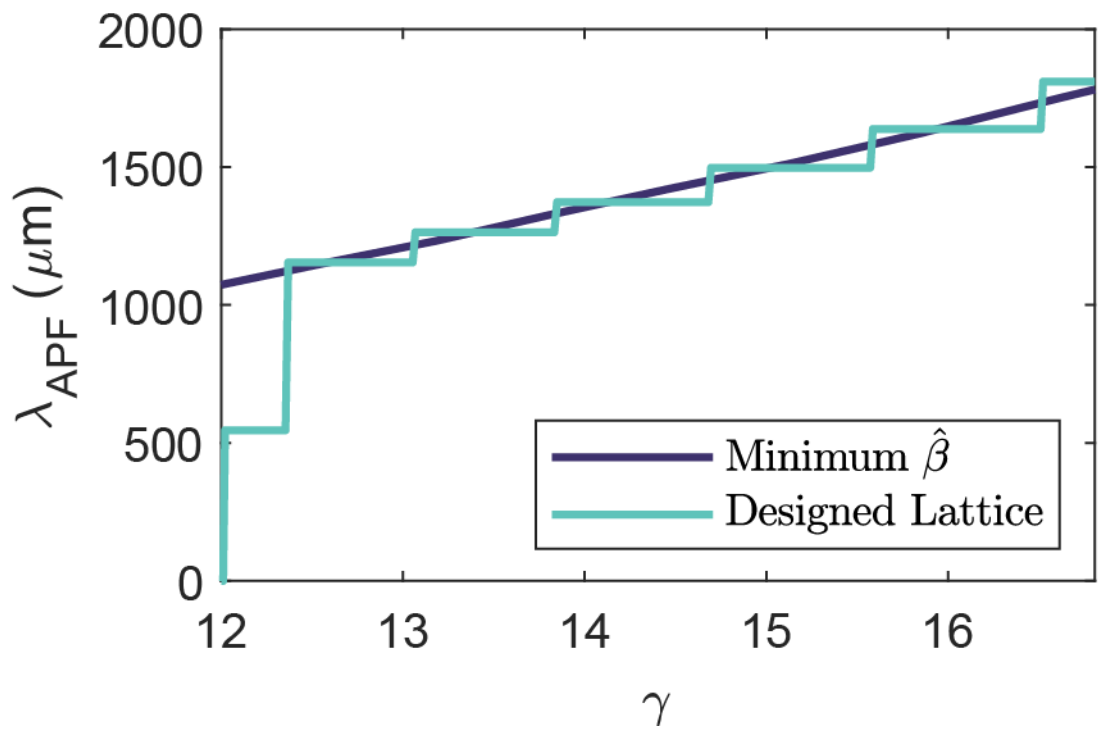

Supplementary Figure 2 APF lattice design to minimize the beam size throughout transport for the $\phi_{APF} = \pi/3$ case.

larger as the beam accelerates. This makes the problem of particle confinement in a small gap more challenging, so we instead simulate a structure with a larger 1.2 μm gap and correspondingly smaller structure factor $\kappa = 0.12$, in accordance with prior structure optimizations [3]. In this case, the beam envelope remains under 500 nm even for $\gamma = 25$, allowing for significant improvement in beam transport.

For each point in Supplementary **??**d, ϕ_{APF} is varied and the parameter λ_{APF} optimize to minimize the maximum of the $\hat{\beta}$ function throughout the DLA. One such example is given in Supplementary Fig. 2 for the $\phi_{APF} = \pi/3$ case; as the beam gains energy the APF lattice period increases, along with the amplitude of the oscillations. Each ϕ_{APF} and associated optimized lattice can then be simulated and energy gain and particle throughput calculated.

The corresponding beam phase space throughout bunching and acceleration is shown in Supplementary Fig. 3. The sinusoidal energy modulation imprinted by the laser is converted into density modulation by velocity dispersion in the drift, resulting in a train of microbunches spaced by the laser wavelength λ_0. The simulation predicts a peak current enhancement corresponding to microbunch widths on the order of hundreds of attoseconds.

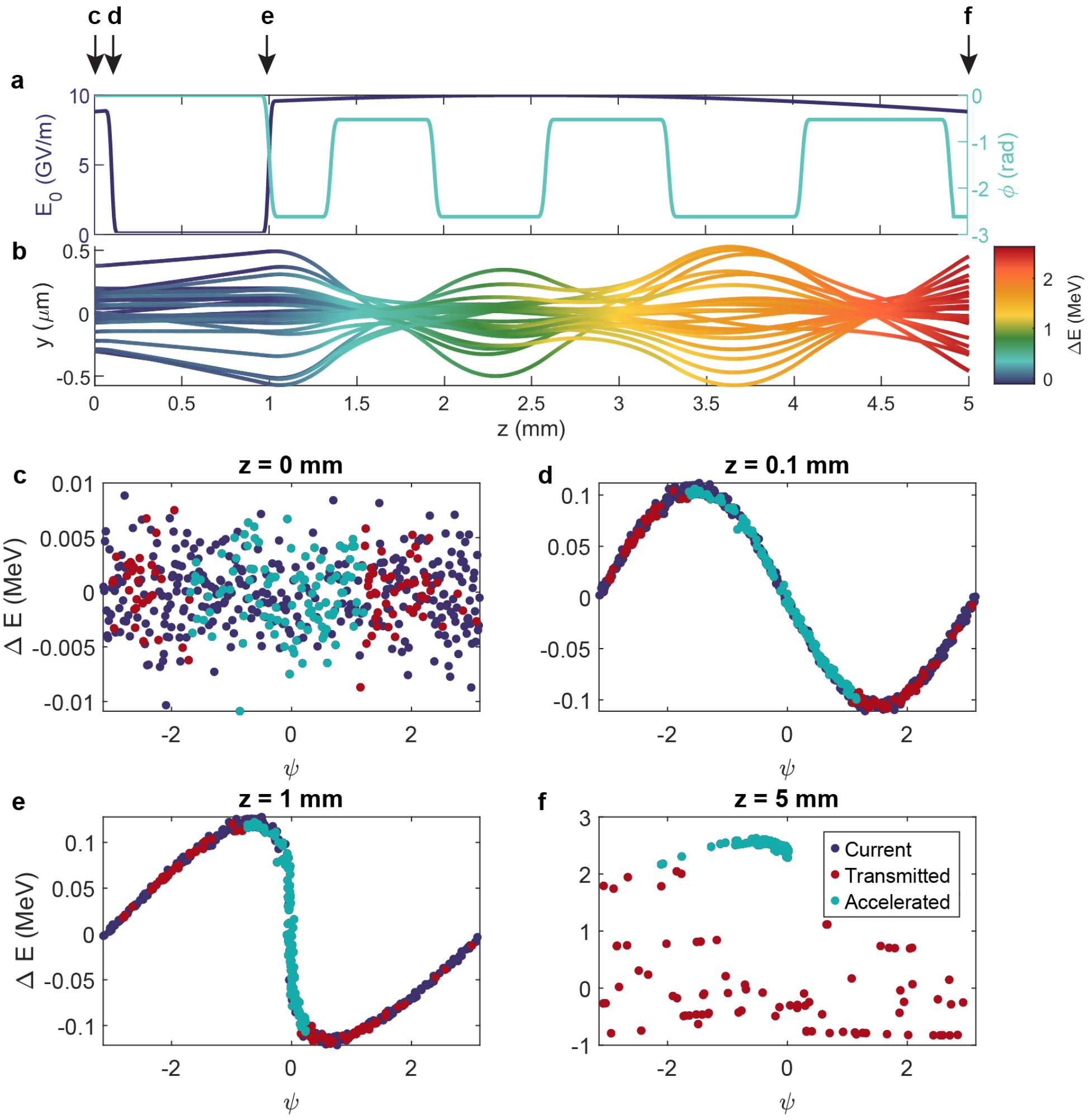

Supplementary Figure 3 **a** Phase and amplitude for APF based buncher and accelerator. **b** Sample particle trajectories, colored according to particle energy. **c,d,e,f** Energy vs longitudinal phase ψ at various points in the prospective APF based design. Particles colored in dark blue are transmitted to that position, but ultimately do not get through the accelerator. Particles colored in red are transmitted, but not accelerated to the intended energy. Particles colored in teal are both transmitted and accelerated. **c** The initial, uniform, distribution. **d** Distribution at the end of the initial driven section, indicating sinusoidal modulation. **e** Distribution after the drift section; the beam has ballistically bunched. **f** Final phase space.

Supplementary References

[1] Naranjo, B., Valloni, A., Putterman, S., Rosenzweig, J.B.: Stable charged-particle acceleration and focusing in a laser accelerator using spatial harmonics. Phys. Rev. Lett. **109**, 164803 (2012) https://doi.org/10.1103/PhysRevLett.109.164803

[2] Niedermayer, U., Egenolf, T., Boine-Frankenheim, O., Hommelhoff, P.: Alternating-phase focusing for dielectric-laser acceleration. Phys. Rev. Lett. **121**, 214801 (2018) https://doi.org/10.1103/PhysRevLett.121.214801

[3] Crisp, S., Ody, A., Musumeci, P.: Asymmetric dual-grating dielectric laser accelerator optimization. Instruments **7**(4) (2023) https://doi.org/10.3390/instruments7040051
