# Supplementary material for: Dynamic control of laser driven electron acceleration in a photonic structure using programmable optical pulses
Source: Nat Commun. 2026 Jul 8;17:8424. doi: 10.1038/s41467-026-75233-z (PMC13478337; doi:10.1038/s41467-026-75233-z)
Supplement: Supplementary file 2 [file 41467_2026_75233_MOESM2_ESM.pdf]

nature portfolio

Peer Review File

Dynamic control of laser driven electron acceleration in a photonic structure using programmable optical pulses

Corresponding Author: Dr Sophie Crisp

This file contains all reviewer reports in order by version, followed by all author rebuttals in order by version.

Version 0:

Reviewer comments:

Reviewer #1

(Remarks to the Author)
I am glad to review the manuscript entitled “Dynamic control of laser driven electron acceleration in a photonic structure using programmable optical pulses” by Sophie Crisp et al. The authors report experimental results applying a spatial light modulator (SLM) in combination with a pulse-front-tilt setup to optimize the temporal overlap of laser and electron beams in a dielectric grating. This approach aims to maximize the energy gain while simultaneously projecting focusing schemes onto a simple periodic grating.

The manuscript presents an important achievement: a record interaction length and the first reported use of an SLM in this context. These results are certainly of interest to the DLA community. However, in its present form, the manuscript raises several issues that should be addressed before it can be considered for publication in Nature Communications.

1. The record interaction length is achieved without the SLM, since—as probably expected—a simple linear phase correction is sufficient to overcome dephasing. This raises the question of whether the SLM is truly required, or whether linear phase correction alone would suffice.

2. The terminology is at times confusing. The phase changed in the APF and bunching measurements is sometimes called $\Delta\Phi_{APF}$, sometimes $\Delta\Phi_{b}$. I understand that this should indicate the purpose of the phase change but it is always the same synchronous phase which is changed. Also the mixing of abbreviations SLM and LCM is problematic for readers not already familiar with these optical elements.

3. It is valuable that the authors discuss some downsides of using an SLM. However, this weakens the central claim that SLMs are necessary for controlling the DLA interaction. In fact, a piezo mirror was used to achieve the record interaction length, and the APF scheme shows more potential than ponderomotive focusing, though the phase jumps in the APF scheme appear too abrupt for SLM modulation.

4. Comments to Fig. 2 and the corresponding part of the manuscript:
a) The plot layout is clear, but the placement of stars at the top of the energy gain lineout makes it difficult to see the functional (possibly linear) dependence between z and the angular correction.
c) If I am not mistaken, the central region is not 1.7 mm as stated in the caption.
e) Is this the spectrum measured with the piezo mirror instead of the SLM? There seems to be a factor of two between the maximum energy gain in plots (d) and (e). Please clarify. Is the angular correction due to the piezo mirror comparable to plot (d)?

5. Comments to Fig. 4 and the corresponding part of the manuscript:
c top) There is a strong asymmetry between gain and loss in the experimental data, which is not reflected in the simulations. Is dephasing mentioned in the text underestimated in the simulations?
c bottom) The simulations used for comparison do not match the experimental data points. Could the discrepancy be due to errors in the phase jump introduced by SLM smoothing? An estimate of this error would be helpful.

6. The manuscript states that the proposed focusing schemes should increase transmission. Unfortunately, transmission is not shown. Including such data would strengthen the claim by showing not only increased energy gain but also improved

confinement.

The potential of using an SLM to control DLA interactions is clear. However, the current experimental data do not yet convincingly support the claim or match all the corresponding simulations. I therefore recommend publication in Nature Communications provided that the authors revise their manuscript to address the points raised above.

Reviewer #2

(Remarks to the Author)
The authors report experimental results for a DLA structure controlled by both linear and second-order (Ponderomotive) laser fields for the acceleration and confinement of high-energy electrons from the Pegasus facility. It is a prime experimental demonstration of the ponderomotive confinement scheme proposed by Naranjo et. al. some 13 years ago. This work is the latest in a series of works by Crisp et. al. and is a wonderful practical example of novel accelerator physics. I fully recommend publication of the manuscript.
The authors have done an excellent job in writing the manuscript and showcasing their data. The text is clear and direct, the flow is well thought-of, and the references are spot-on. I only have a number of short comments and some questions, listed below.

Questions relating to the main text:
1. The calibration method described in lines 159-162 and Figure 2a only works if the velocity of the electrons does not change appreciably when accelerating from the entrance to the exit of the structure. It would strengthen this claim if the authors showed a simple calculation of the phase mismatch accumulated due to the 0.55 MeV acceleration, and could be used to predict to what energy can one accelerate using this kind of calibration before appreciable mismatch sets in.
2. In Fig.3a, I would expect the intensity around the optical axis in the x-z plane to be mirror-symmetric around x, because the structure is symmetric. But it does not seem symmetric from the figure, why?
3. In the Ponderomotive focusing starting on line 205: what is the role of the periodicity of the grating itself in the Ponderomotive scheme? How does it relate to Ap, Tp ?
4. In line 266 the authors claim they can ballistically-bunch the beam. They repeat this by mentioning simulations in line 277. This is very interesting: How well can you ballistically-bunch electrons at these energies without traditional methods (e.g. a chicane)? Could you add to the methods a section or supplementary these mentioned simulations, showing the bunches and indicating their width, and add a clear reference to them in the main text? (If I understood correctly, Fig.1 of the supplementary is for the ponderomotive scheme or the combined schemes).

I have a number of questions about the SLM, which I think are important to point out for anyone wishing to reproduce this method:
5. One question that did not arise in the manuscript and should be addressed, is the question of the twin-image problem. The scheme used in this work is in-line holography, which means that both real and virtual images are overlapping. Has this been considered?
6. Another caveat of in-line holography is longitudinal diffraction orders, e.g. in the case of lines 126-127 with the example of the variable Fresnel lens. Also depending on the bandwidth of the laser pulses, each wavelength will deviate from the nominal focal length. Is this negligible? 1-2 sentences supported by a back-of-the-envelope calculation could be inserted around those lines.
7. Lastly, when using the SLM, I would expect the edges of the LC pixels to diffract some photons. This would create unavoidable diffraction orders at what should be relatively high angles, particularly in the example in lines 126-127, where one axis is still imaging the SLM and the other is the conjugate plane. The authors allude to this in lines 418-420 referring to "the first order reflection", which is slightly confusing: do you imply using off-axis holography? Or do you mean the first-order diffraction that is superimposed on all other orders, by virtue of on-axis (in-line) holography? (Note that I don't mean the phase discontinuities apparent in the imaged plane as elaborated on in line 215 and Methods 4.4, but the conjugate or Fourier plane).

Additional comments:
- Line 21: "..the demonstration *of* software-based.."
- Line 124: LCM is first used, but not defined. I suggest to replace all instances of "LCM" with "SLM" (except perhaps in the methods, if you wish to go into details of the SLM).
- Line 178: did you mean "reflection losses"? You previously mentioned that the SLM is reflective.
- Line 250: "that *the* field experienced"
- Line 326: remove one "the"
- Line 330: units of dispersion in cm?
- Line 345 vs 347: is n=0 "mode" or "peak"?
- The caption of Fig.4 mentions "gamma_res" and "Delta E_res", but these are not defined in the text.
- Also in Fig.4, there are curves for "Gain" and "Loss" but I am unclear as to the exact definition of these. Are these measured from the nominal (injection) energy to the highest/lowest measured electron energy? Please define them.
- In your discussion, lines 296-297, please note the nominal (injection) energy used for these simulations here.

Reviewer #3

(Remarks to the Author)

This manuscript reports on dynamic control of relativistic electron acceleration in double-grating structures. The authors employ spatial light modulators (SLM) to tailor incident light phases and amplitudes, thereby enabling phase-matching, pondermotive focusing, and alternating phase focusing (APF). This approach differs from conventional methods that rely on photonic nanostructures fabrication, offering a potentially more flexible and convenient way to manipulate both the transverse and longitudinal dynamics of electron acceleration.
The concept is interesting and could represent an important step toward versatile control of electron acceleration in DLA. However, based on the data currently presented, the advantages of beam control using SLM are not sufficiently clear. Several key issues should be addressed to strengthen the manuscript.
1. The authors claim 0.55 MeV energy gain, but this energy gain is not observed by using SLM. What's the laser damage threshold of SLM?
2. In fig.3, only maximum energy gains are present. One can conclude less energy-gain with the sinusoidal modulation on SLM. However, to demonstrate the pondermotive focusing, more data should be provided, such as the full electron energy spectra, the images of transverse electron distributions, the changes of output electron current.
3. Similarly, in the demonstration of APF, only maximum energy gains are presents. Could authors provide more experimental data to support APF?
More detailed comments below:
4.The electron microscopy image of DLA structure is missing.
5. Fig.2a presents the incident-angle dependence of the maximum electron energy gain. However, the use of polylines in the plots obscures the underlying experimental data, making it difficult to evaluate the results directly. It would be helpful if the authors could show the raw data points more clearly. In addition, an explanation why the maximum energy gain is dependent on z location is missing.
6. The z location of fig.2c is ~3.4mm, however, the z location of corresponding laser profiles in fig.2b is 4.25mm, since it corresponds to the second lineout in firg.2a.
7. In fig. 3b, the simulations show the electrons gain the same energy at $\Delta\theta=0$. However, the interaction length varies from 0.5mm to 5mm. Does the intensity of light field is also changed?
8. In fig. 3d, the meaning of the dashed line is not explained. The authors should clarify what this line represents in the figure.
9. In the line 223, how to obtain the 'effective interaction length 2.5±0.5mm' from those $|\sin\Delta\theta|$ function-liked curves?
10. The light intensity directly affects the electron transverse focusing length and drift length. In fig. 4d, I_0 is arbitrary units, what is the absolutely value of light field?
11. It is not clear whether the use of a pulse-front-tilted laser has any influence on the spatial modulation introduced by SLM. The authors should clarify if and how pulse-front tilt affects the effectiveness or accuracy of the phase and amplitude control.

Version 1:

Reviewer comments:

Reviewer #1

(Remarks to the Author)
I am glad to review the revised manuscript entitled “Dynamic control of laser driven electron acceleration in a photonic structure using programmable optical pulses” by Sophie Crisp et al. I would like to thank the authors for the very detailed reply to the reviewers' comments and the changes made in the manuscript to address the reviewers' concerns. Based on these changes and the additional explanations in the authors' reply to the reviewers I fully recommend publication of the revised manuscript in Nature Communications.

Reviewer #2

(Remarks to the Author)
The authors' rebuttal addresses my concerns fully. Good job!

Reviewer #3

(Remarks to the Author)
The manuscript presents an original and well-motivated contribution to laser acceleration in a photonic structure, using sound methodology to modulate the light phase. The authors have addressed major concerns about the advantages of application of spatial light modulator in ponderomotive focusing and alternate phase focusing. The additional data presentation and text explanation make the statements clear and convincing. Overall, I recommend it for publication.

Open Access This Peer Review File is licensed under a Creative Commons Attribution 4.0 International License, which permits use, sharing, adaptation, distribution and reproduction in any medium or format, as long as you give appropriate credit to the original author(s) and the source, provide a link to the Creative Commons license, and indicate if changes were made.
In cases where reviewers are anonymous, credit should be given to 'Anonymous Referee' and the source.
The images or other third party material in this Peer Review File are included in the article's Creative Commons license, unless indicated otherwise in a credit line to the material. If material is not included in the article's Creative Commons license and your intended use is not permitted by statutory regulation or exceeds the permitted use, you will need to obtain permission directly from the copyright holder.
To view a copy of this license, visit https://creativecommons.org/licenses/by/4.0/

Reviewer #1: I am glad to review the manuscript entitled "Dynamic control of laser driven electron acceleration in a photonic structure using programmable optical pulses" by Sophie Crisp et al. The authors report experimental results applying a spatial light modulator (SLM) in combination with a pulse-front-tilt setup to optimize the temporal overlap of laser and electron beams in a dielectric grating. This approach aims to maximize the energy gain while simultaneously projecting focusing schemes onto a simple periodic grating.

The manuscript presents an important achievement: a record interaction length and the first reported use of an SLM in this context. These results are certainly of interest to the DLA community. However, in its present form, the manuscript raises several issues that should be addressed before it can be considered for publication in Nature Communications.

1. The record interaction length is achieved without the SLM, since—as probably expected—a simple linear phase correction is sufficient to overcome dephasing. This raises the question of whether the SLM is truly required, or whether linear phase correction alone would suffice.

We thank the reviewer for this insightful comment. We agree that it is important to clarify why the SLM remains a critical component of the experimental setup.

First, regarding the terminology, we wish to clarify that the "simple linear phase correction" mentioned by the reviewer refers to a linear variation in the incidence angle, $\theta_I(z)$. In terms of the optical wavefront, this actually corresponds to a quadratic phase profile (a curved wavefront), rather than a linear phase ramp.

Second, we would like to make a distinction regarding the experimental results: while the *record energy gain* was indeed obtained using the piezo mirror, this was achieved by enabling higher laser intensity (fluence), not by achieving a *record interaction length* than the SLM. In fact, the SLM was strictly necessary to establish that interaction length in the first place. The piezo mirror was only able to be used effectively because the SLM had already been allowed to determine that the required correction happened to be quadratic.

We have revised Section 2 (Results) to explicitly clarify the distinction between linear angular correction and quadratic phase, and to emphasize that the mirror substitution was a specific optimization strategy enabled by the SLM's initial characterization.

2. The terminology is at times confusing. The phase changed in the APF and bunching measurements is sometimes called $\Delta\Phi_{APF}$, sometimes $\Delta\Phi_b$. I understand that this should indicate the purpose of the phase change but it is always the same synchronous phase which is changed. Also the mixing of abbreviations SLM and LCM is problematic for readers not already familiar with these optical elements.

We have removed all references to LCMs and replaced them with SLM for clarity.

3. It is valuable that the authors discuss some downsides of using an SLM. However, this weakens the central claim that SLMs are necessary for controlling the DLA interaction. In fact, a piezo mirror was used to achieve the record interaction length, and the APF

scheme shows more potential than ponderomotive focusing, though the phase jumps in the APF scheme appear too abrupt for SLM modulation.

We thank the reviewer for this comment. We believe the intent of the paper is to clearly present the advantages as well as the lessons learned from using an SLM-based tuning scheme. We acknowledge that the damage threshold and pixel discretization of the SLM limited the maximum gradient compared to static optics. However, we maintain that the SLM is necessary for continued gains because it provides a degree of fine-tuning and flexibility that static elements cannot offer. We view the SLM as the optical equivalent of mechanical tuners or low-level RF control in conventional copper accelerators: in an ideal scenario a fixed phase profile might suffice. However, experimental imperfections (wavefront aberrations, fabrication errors, alignment drifts) are unavoidable, and the SLM is essential precisely because it allows us to compensate for these imperfections *in situ*.

Furthermore, the SLM transforms the accelerator into a programmable device. It allows us to tune on demand, dynamically switching between configurations optimized for high transmission, maximum energy gain, or bunching without changing the hardware. While we envision that these "control knobs" will eventually be integrated directly on-chip (e.g., via integrated phase shifters), the SLM currently serves as an indispensable tool for identifying the control laws required for that future integration.

As for APF vs. ponderomotive focusing we have tried in this paper to steer out of the question which scheme is better. In the literature it is often mentioned as APF is a better candidate for lower energy focusing, but at higher energy ponderomotive focusing has more favorable scaling, so it is likely that depending on the final energy of the DLA a mixture of the schemes should be employed. Here the SLM allows us to test some important characteristics of both schemes in the same setup.

We amended the manuscript to reflect this point better:

"In fact, while static optics may offer higher power handling, the SLM provides a critical layer of active feedback necessary to correct for inevitable experimental imperfections in the laser wavefront, structure fabrication, and alignment. This methodology allows for the manipulation of the electron phase space without the need for precise *a priori* knowledge of these errors, effectively enabling 'live' tuning of the matched phase irrespective of the specific cause of dephasing."

4. Comments to Fig. 2 and the corresponding part of the manuscript:
a) The plot layout is clear, but the placement of stars at the top of the energy gain lineout makes it difficult to see the functional (possibly linear) dependence between z and the angular correction.

We have added dots on each line to more clearly show the underlying data. We note again that the approximately linear relation between position and angle corresponds to a quadratic phase correction.

c) If I am not mistaken, the central region is not 1.7 mm as stated in the caption.

Referee is correct. We amended the caption.

e) Is this the spectrum measured with the piezo mirror instead of the SLM? There seems to be a factor of two between the maximum energy gain in plots (d) and (e). Please clarify. Is the angular correction due to the piezo mirror comparable to plot (d)?

We have amended the caption to clarify that yes, the spectrum of (e) is obtained with the SLM replaced with the piezo mirror for maximal modulation to avoid the limitations (losses and damage threshold) associated with using the SLM. As is explained in the text, we found that there were some optimal SLM tunings which corresponded to quadratic phase fronts, which could be fixed by translating the lenses in the laser transport.

5. Comments to Fig. 4 and the corresponding part of the manuscript:
c top) There is a strong asymmetry between gain and loss in the experimental data, which is not reflected in the simulations. Is dephasing mentioned in the text underestimated in the simulations?
c bottom) The simulations used for comparison do not match the experimental data points. Could the discrepancy be due to errors in the phase jump introduced by SLM smoothing? An estimate of this error would be helpful.

We thank the referee for this detailed observation. The asymmetry between gain and loss is very common whenever the resonance condition has not been perfectly tuned (by adjusting the incident laser angle or in our case the SLM linear angle ramp). This was already noted and observed in [12] D. Cesar et al. , Optics Express 2018.

Regarding the discrepancy between simulation and data in Fig. 4c (bottom), we agree that this is likely due to errors introduced by the SLM at the phase jumps. As the reviewer suggests, the SLM cannot produce an infinitely sharp step function; there is inherent smoothing due to point spread function of the optical system, pixel crosstalk and liquid crystal response. Furthermore, sharp phase transitions often result in localized intensity dips due to diffraction effects that are difficult to model perfectly. While these effects tend to "wash out" the sharp features of the modulation, we can observe distinctly a clear shift in the energy spectrum compared to the zero drift case which is a robust signature of the underlying beam dynamics we aimed to demonstrate.

We propose adding a clarifying sentence to the Results section where Fig. 4 is discussed.

“Comparison with simulations indicate that the observed shift is due to an induced sub-wavelength density modulation. We attribute the reduced modulation depth in the experimental data compared to simulations to the finite resolution of the SLM, which introduces phase smoothing and intensity gaps at the sharp transition points required by this scheme."

6. The manuscript states that the proposed focusing schemes should increase transmission. Unfortunately, transmission is not shown. Including such data would strengthen the claim by showing not only increased energy gain but also improved confinement.

We agree with the reviewer that demonstrating increased transmission would be the ideal direct evidence for improved confinement. Unfortunately, in the current experimental setup, evidence for focusing is strongly suppressed by the fact that not all the electrons in the beam experience the laser field, and ultimately completely obscured by the large instability in transmitted beam charge.

Specifically, the temporal overlap between the electron bunch (~ 1 ps) and the laser pulse (~ 100 fs) means that at most 10% of the electron charge interacts with the focusing field. Simulations for the parameters used in the experiment predict at best a transmission enhancement of <10% for the interacting slice.

However, experimentally, we observe shot-to-shot transmission fluctuations of approximately ±12\%, driven by incoming charge stability (2.4 ±0.2 pC as measured by an upstream toroid) coupled to pointing and energy jitter which decorrelate the transmission from the input charge (linear fit R^2 ~0.41). Consequently, the predicted small focusing effect on a thin slice of the beam is entirely buried within the baseline noise of the unmodulated majority of the beam.

While we could not directly measure the integrated current increase, the angular scan data for the ponderomotive sinusoidal phase modulation (Fig. 3) confirm that many non resonant modes were successfully excited. Since the longitudinal and transverse forces are coupled, the observation of the characteristic energy modulation signatures provides indirect but physically robust evidence that the corresponding focusing forces were present, even if the integrated transmission signal was below the detection threshold.

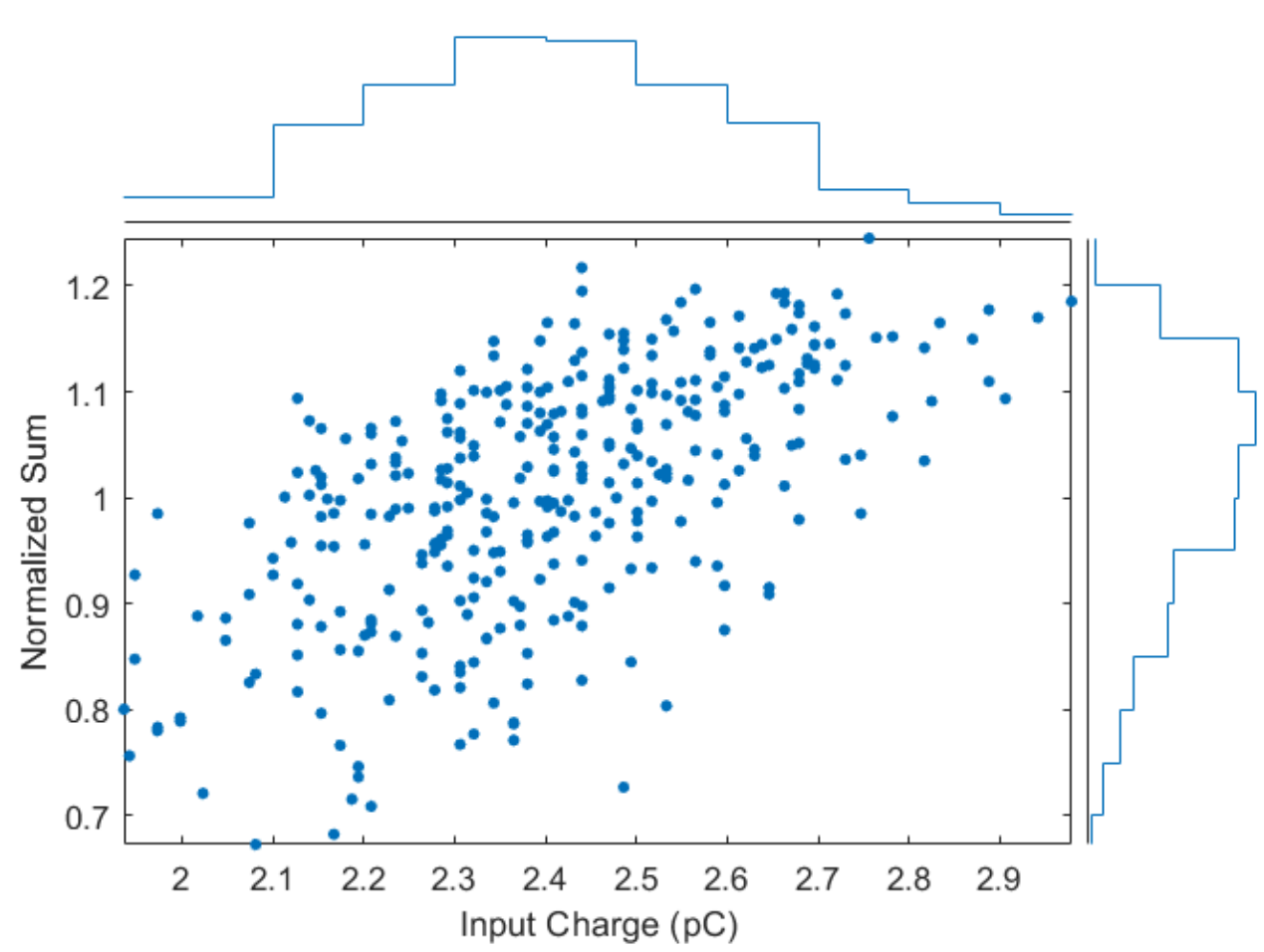

Figure 1. Transmitted beam intensity (measured as the sum of pixel counts from the spectrometer camera vs. input beam charge. The small correlation is not sufficient to eliminate the noise in the transmission data.

The paper has been amended to explain better this point

“In the experiment, due to pointing jitter and the mismatched phase space of the incoming e-beam, particle throughput was highly variable; consequently, we were not able to distinguish the relatively small expected increase in integrated current from the larger shot-to-shot charge fluctuations of the portion of the electron beam not temporally overlapping with the laser. Nevertheless, we observed clear signatures of both of these focusing schemes in the energy spectra of the beams, providing insights into the underlying beam dynamics.“

We go in more details on the observed fluctuations in the electron beam section of the methods:
Electron Beam Measurements

“An Integrating Current Transformer (ICT) was used to measure the input charge as having $\approx12\%$ fluctuations; comparison of the measured input charge and transmitted charge indicate that pointing and energy jitter decouple these variables (linear fit $R^2 = 0.41$). As a result, we normalize all spectra to the transmitted, not inputted, charge, allowing us to make direct comparisons of laser off-laser on shots to extract energy gain/loss without the influence of charge variations’”

The potential of using an SLM to control DLA interactions is clear. However, the current experimental data do not yet convincingly support the claim or match all the corresponding simulations. I therefore recommend publication in Nature Communications provided that the authors revise their manuscript to address the points raised above.

Reviewer #2:

The authors report experimental results for a DLA structure controlled by both linear and second-order (Ponderomotive) laser fields for the acceleration and confinement of high-energy electrons from the Pegasus facility. It is a prime experimental demonstration of the ponderomotive confinement scheme proposed by Naranjo et. al. some 13 years ago. This work is the latest in a series of works by Crisp et. al. and is a wonderful practical example of novel accelerator physics. I fully recommend publication of the manuscript.

The authors have done an excellent job in writing the manuscript and showcasing their data. The text is clear and direct, the flow is well thought-of, and the references are spot-on. I only have a number of short comments and some questions, listed below.

Questions relating to the main text:

1. The calibration method described in lines 159-162 and Figure 2a only works if the velocity of the electrons does not change appreciably when accelerating from the entrance to the exit of the structure. It would strengthen this claim if the authors showed a simple calculation of the phase mismatch accumulated due to the 0.55 MeV acceleration, and could be used to predict to what energy can one accelerate using this kind of calibration before appreciable mismatch sets in.

We fully agree with the reviewer that the calibration method relying on piecewise optimization is strictly valid only when the velocity change within the interaction window is small (the ballistic limit).

To quantify this, we use the dephasing length approximation derived by Breuer et al. (*J. Phys. B: At. Mol. Opt. Phys.* 47, 234004, 2014), defined as the distance over which the phase mismatch reaches pi/2.

$$L_{deph} = \sqrt{\frac{\lambda_0 mc^2 \beta^3 \gamma^3}{4G_{max}}}$$

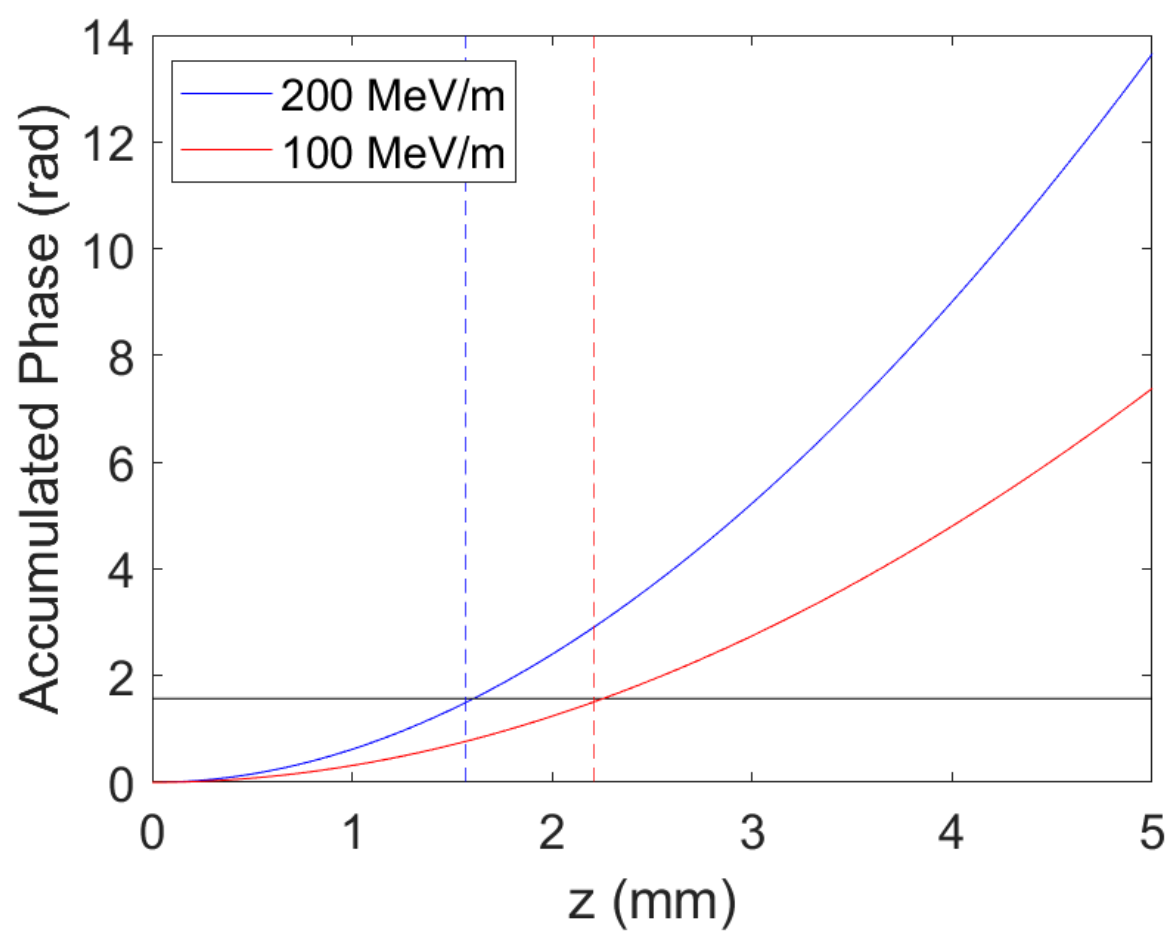

Figure 2. For our experimental parameters (λ_0 = 780 nm and total energy of 8.7 MeV), assuming an accelerating gradient of 200 MeV/m as achieved in the piezo-mirror case, the dephasing length is approximately 1.6 mm. For the 100 MeV/m maximal gradient that could be achieved with the SLM, the interaction extends to 2.2 mm.

Two factors ensured the validity of our calibration. First, during the SLM calibration scans, the effective gradient was significantly lower than 200 MeV/m due to the optical losses described in the Methods (SLM reflectivity, diffraction efficiency, etc.). Second, the calibration relies on optimizing the phase for a focused laser spot with an effective interaction extent of approximately 1.7 mm. Since this distance is well below the calculated dephasing length, the velocity change within the calibration window was negligible, and the ballistic assumption held.
We note that when operating in a strongly dynamic regime (high gradient over long distances), the SLM could be used to eliminate this dynamic phase slippage by adding a compensating phase profile ϕ_{dyn}= k_0∫ (1 - 1/□(z)) dz, where □(z) is derived from the integrated energy gain.

2. In Fig.3a, I would expect the intensity around the optical axis in the x-z plane to be mirror-symmetric around x, because the structure is symmetric. But it does not seem symmetric from the figure, why?

This is a good observation. Following this comment, we performed wavefront propagation simulations to isolate the source of the asymmetry in the x-z intensity plane. We found that the observed effect is likely due to a combination of a minor misalignment of the laser with respect

of the axis of the digital Fresnel lens applied to the SLM and an outdated voltage-to-phase calibration resulting in an insufficient modulation depth to cover the full 0-2π range in applied phase. As the reviewer noted, the structure should be symmetric. However, any offset between the center of the applied digital lens profile and the optical axis of the incident laser beam translates to a transverse shift in the focal plane. When combined with the varying longitudinal phase modulation, this manifests as the observed asymmetry or "wobble" in the x-position.

Crucially, however, our simulations indicate that while the intensity distribution is perturbed by these artifacts, the phase experienced by the electrons along the propagation axis remains closely related to the intended phase profile applied at the SLM. Therefore, despite the visual asymmetry in the laser intensity envelope, the longitudinal phase dynamics required for the ponderomotive and APF schemes are preserved.

We added a clarification sentence in the caption of the Figure.

“Laser profile (upper) and corresponding phase profile (lower) across the structure for the $\Delta\theta=-2$ mrad... (Note: Simulations indicate that the transverse asymmetry in the laser intensity profile is an artifact resulting from a minor misalignment of the SLM-applied Fresnel lens combined with a limited phase modulation range.)”

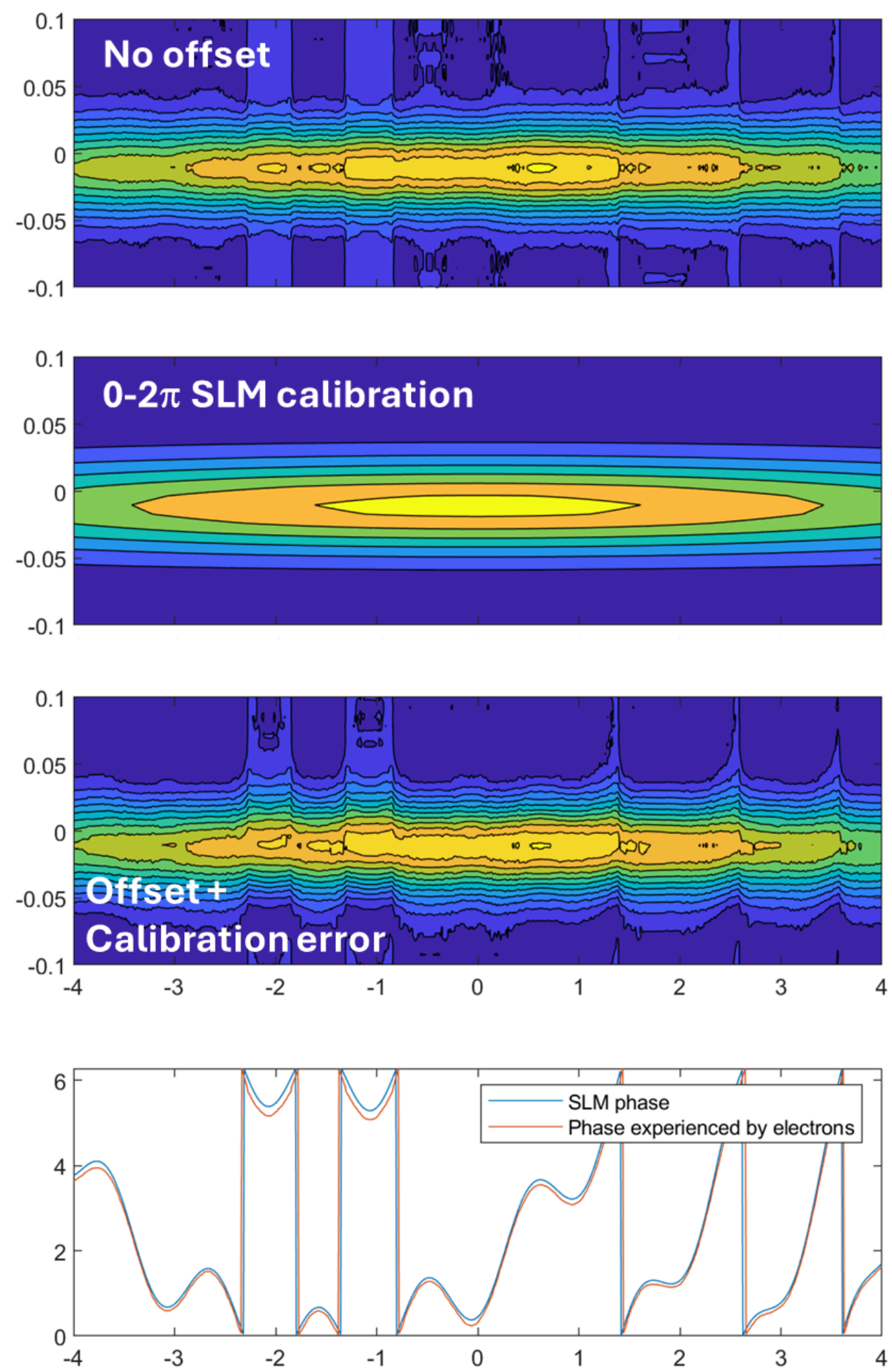

Figure 3: Intensity profile at the DLA plane when the incoming beam has an offset with respect to the SLM Fresnel lens and the SLM is not exactly calibrated to impart a 0-2pi phase shift. The intensity profile when the beam is aligned to the SLM and when an updated calibration is used are also shown for comparison.

3. In the Ponderomotive focusing starting on line 205: what is the role of the periodicity of the grating itself in the Ponderomotive scheme? How does it relate to Ap, Tp ?

We thank the reviewer for asking to clarify the relationship between the fixed structure geometry and the parameters of the applied optical phase modulation.

The periodicity of the DLA grating λ_g defines the spatial periodicity of the wave in the structure and therefore the resonant condition in the absence of any drive laser modulation (that is for a flat incoming phase front).

The DLA ponderomotive focusing scheme relies on the interaction between the electron and non-resonant spatial harmonics. In our setup, these additional harmonics are not generated by

the grating structure itself (which has a fixed period λ_g), but are synthesized optically using the SLM. When applying a sinusoidal phase modulation to the laser drive with amplitude A_p and period T_p, we effectively split the single grating mode into a spectrum of sidebands. According to the Jacobi-Anger expansion this additional phase modulation causes the field experienced by the electron to become a sum of spatial harmonics with wavenumbers $k_n = k_g + n\ 2\pi\ /T_p$ where the modulation period T_p sets the spectral spacing (or "detuning") of the generated sidebands relative to the “carrier” grating wavenumber. In this picture, the modulation amplitude A_p controls the power distribution among these spatial harmonics (scaling as Bessel functions $J_n(A_p)$) thereby determining the relative strength of the resonant accelerating field versus the non-resonant focusing fields.

As discussed in the cited works of Naranjo and Cesar, the result of averaging out the fast non resonant oscillations (when $T_p \gg \lambda_{g)}$ is an effective focusing force on the secular transverse motion of the particles inside the DLA. While the strength of the focusing increases for longer T_p, the spectral spacing between the harmonics decreases eventually resulting in unstable longitudinal motion.

The text has been revisited to better explain the relationship between the parameters of the ponderomotive focusing scheme.

4. In line 266 the authors claim they can ballistically-bunch the beam. They repeat this by mentioning simulations in line 277. This is very interesting: How well can you ballistically-bunch electrons at these energies without traditional methods (e.g. a chicane)? Could you add to the methods a section or supplementary these mentioned simulations, showing the bunches and indicating their width, and add a clear reference to them in the main text? (If I understood correctly, Fig.1 of the supplementary is for the ponderomotive scheme or the combined schemes).

We thank the reviewer for highlighting this point. The ability to ballistically bunch electrons without a magnetic chicane relies on the fact that, at the energies used for this specific measurement (6.2 MeV), the electron beam is relativistic but not ultra-relativistic. In this regime, a short drift space provides a longitudinal dispersion $R\_56 = L / 2\gamma^2$ sufficient to convert energy modulation into density modulation.

We lowered the beam energy from 8.7 MeV to 6.2 MeV for the bunching dataset precisely to enhance this effect. As established in the "attosecond DLA" literature (e.g., Refs [6], [7], [22]), a periodic energy modulation imprinted by the laser followed by a sufficiently long drift allows for the formation of a train of attosecond microbunches. In our setup, assuming a modulation amplitude dg/g of 2%, the drift length required to maximize the bunching factor (R_56 dg/g = pi) is on the order of millimeters, which fits within our experimental capabilities.

Per the reviewer's suggestion, we have added a new section and figure to the **Supplementary Material**. This figure presents the simulation results for the APF 6.2 MeV case, displaying the longitudinal phase space evolution and the resulting current profile, which exhibits distinct microbunches with attosecond-scale widths.

The main text has been revised as follows

"Comparison with simulations (see Supplementary and Supplementary Fig. S3) indicate that this shift is due to an induced sub-wavelength density modulation. By operating at a lowered beam energy of 6.2 MeV, the velocity dispersion in the drift section is sufficient to ballistically bunch the beam into a train of attosecond microbunches, similar to schemes reported in [6, 7]. This structure could then be exploited for significantly higher quality output electron beams with decreased energy spread."

The following text has been added to the supplementary, as well as the figure below:

“The corresponding beam phase space throughout bunching and acceleration is shown in Fig. \ref{fig:apf_buncherDist}. The sinusoidal energy modulation imprinted by the laser is converted into density modulation by velocity dispersion in the drift, resulting in a train of microbunches spaced by the laser wavelength λ0. The simulation predicts a peak current enhancement corresponding to microbunch widths on the order of hundreds of attoseconds.”

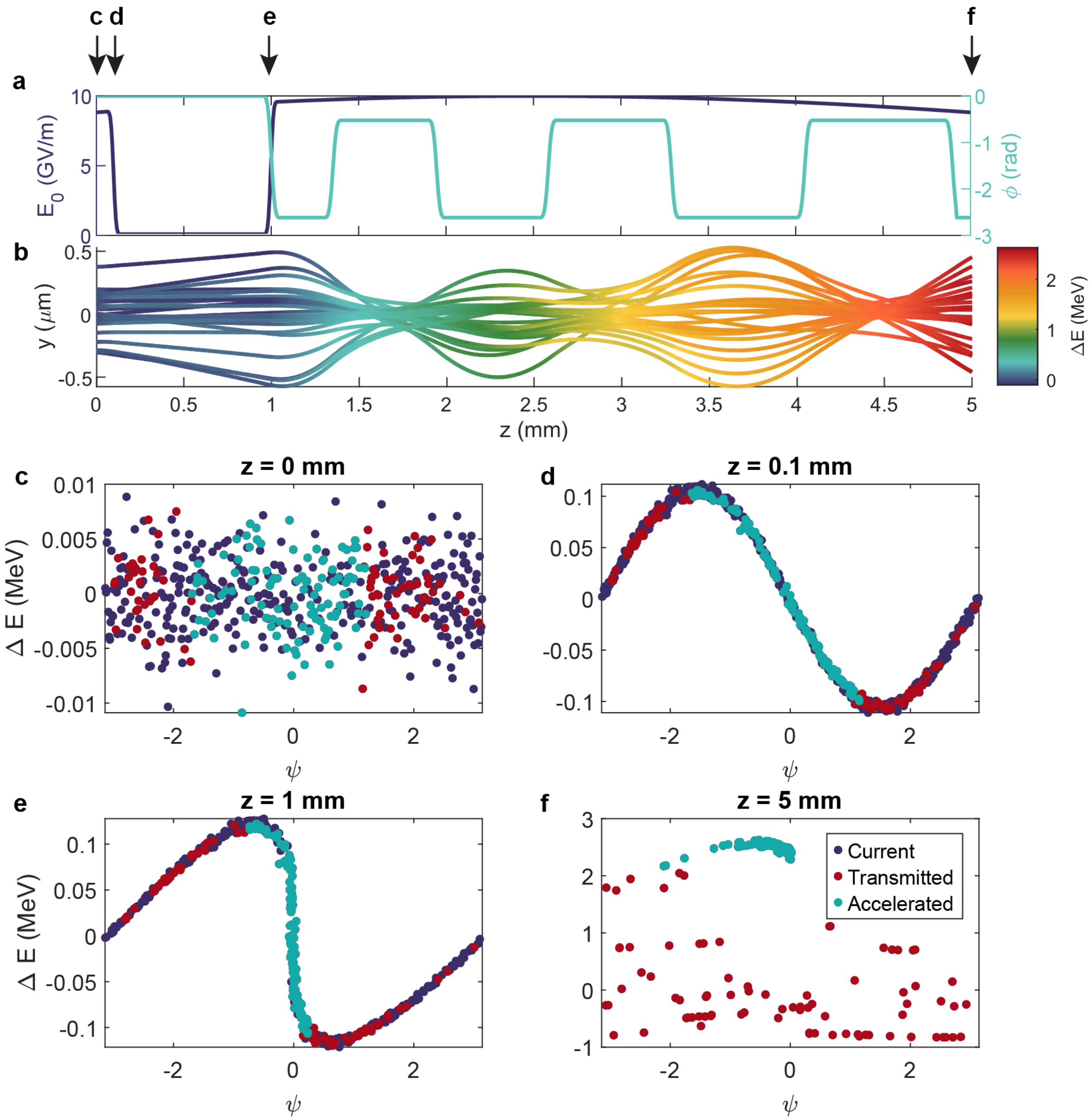

Figure 4. (a) Phase and amplitude for APF based buncher and accelerator. (b) Sample particle trajectories, colored according to particle energy. (c,d,e,f)Energy vs longitudinal phase ψ at various points in a prospective APF based design. Particles colored in dark blue are transmitted to that position, but ultimately do not get through the accelerator. Particles colored in red are transmitted, but not accelerated to the intended energy. Particles colored in teal are both transmitted and accelerated. (c) The initial distribution. (d) Distribution at the end of the initial driven section. (e) Distribution after the drift section. (f) Final phase space.

I have a number of questions about the SLM, which I think are important to point out for anyone wishing to reproduce this method:

5. One question that did not arise in the manuscript and should be addressed, is the question of the twin-image problem. The scheme used in this work is in-line holography, which means that both real and virtual images are overlapping. Has this been considered?

In our SLM-driven laser modulation experiments, we mitigate the twin image problem by adding a linear phase ramp. Experimentally, we noticed that when phase matching was accomplished at zero angular tilt from the SLM ramp, the results were worse, likely due to a combination of the SLM reflection and the twin image problem. The solution we implemented was optical (mis)alignment (and slight angular detuning of the SLM) so that the unwanted twin is not superimposed at the interaction region. Adding a few mrad linear phase ramp ensured that the phase-front shaping is faithful, delivering the intended optical field to the electrons without a distorted duplicate image blurring it. We added a sentence in the methods section of the SLM to clarify this.

6. Another caveat of in-line holography is longitudinal diffraction orders, e.g. in the case of lines 126-127 with the example of the variable Fresnel lens. Also depending on the bandwidth of the laser pulses, each wavelength will deviate from the nominal focal length. Is this negligible? 1-2 sentences supported by a back-of-the-envelope calculation could be inserted around those lines.

Ultrafast Ti:sapphire lasers have a finite bandwidth (~10 nm FWHM at 780 nm), meaning the beam is not strictly monochromatic. This spectral width can lead to longitudinal chromatic aberration as different wavelengths focus at slightly different distances. The magnitude of this focal shift depends on the focusing optics: for a diffractive optic like the SLM-programmed Fresnel lens the focal length is proportional to wavelength, and a ~1.3% bandwidth (10 nm of 780 nm) yields roughly a 1.3% variation in focal distance. Back-of-the-envelope: for an SLM-based optical system programmed to focus with an effective focal length of 200 mm for $\lambda_0 = 780$ nm, the 775 nm components would focus at about 198.7 mm and 785 nm at ~201.3 mm – a spread of about 2.6 mm in focal position. Such shifts could be significant relative to typical depth-of-focus and degrade beam quality if not managed. In our system, the impact is mitigated by the fact that the depth of focus of our beam is fairly large compared to the shift. The Rayleigh range of the ultrafast beam in our experiment is on the order of a few cm, comfortably exceeding the 2 mm-scale chromatic focal variation, so all wavelength components still overlap at the DLA within the interaction length.

We added a sentence in the methods to clarify the minimal effects of the chromaticity of the Fresnel lens

7. Lastly, when using the SLM, I would expect the edges of the LC pixels to diffract some photons. This would create unavoidable diffraction orders at what should be relatively high angles, particularly in the example in lines 126-127, where one axis is still imaging the SLM and the other is the conjugate plane. The authors allude to this in lines 418-420 referring to "the first order reflection", which is slightly confusing: do you imply using off-axis holography? Or do you mean the first-order diffraction that is superimposed on all other orders, by virtue of on-axis (in-line) holography? (Note that I don't mean the phase

discontinuities apparent in the imaged plane as elaborated on in line 215 and Methods 4.4, but the conjugate or Fourier plane).

Our optical setup utilizes astigmatic imaging: one axis is relay-imaged to the DLA, while the other is focused via a cylindrical lens. In the imaged axis, the relay optics resolution limit smooths out pixelation artifacts, while in the focused axis, pixel diffraction generates weak satellite spots that fall outside the DLA channel due to the high fill factor and low numerical aperture. We emphasize that our astigmatic imaging design was partly motivated to allow amplitude modulation by adjusting the Fresnel lens on the SLM, but it also has the side benefit of reducing pixelation artifacts: by not imaging the SLM in one axis, we avoid directly projecting the pixel structure onto the interaction plane. The impact of pixel-boundary diffraction in our setup thus boils down to a slight efficiency loss (a few % of light in undesired orders) and a low-intensity residual on-axis light.

We mitigate the unmodulated zero-order reflection by slightly tilting the SLM and using spatial filtering to direct only the intended "first-order" modulated beam into the accelerator. It's important to distinguish the pixel-boundary diffraction from the intentional “first-order” diffraction used to form the holographic pattern. The first-order beam in our setup refers to the SLM-modulated beam containing the encoded phase profile which is the beam we image onto the DLA. The zero-order refers to the specular reflection/unmodulated light from finite fill factor or incomplete phase wrapping, which we try to remove using a slight SLM tilt plus spatial filtering so that the first order diffraction is the one directed into the accelerator. Consequently, we observe no "twin-image" artifacts carrying spurious phase patterns, but only a negligible diffuse background.

Additional comments:
*- Line 21: "..the demonstration *of* software-based.."*

Corrected.

- Line 124: LCM is first used, but not defined. I suggest to replace all instances of "LCM" with "SLM" (except perhaps in the methods, if you wish to go into details of the SLM).

We have removed all instances of ‘LCM’ and replaced with ‘SLM’

- Line 178: did you mean "reflection losses"? You previously mentioned that the SLM is reflective.

Yes, corrected.

*- Line 250: "that *the* field experienced"*

Corrected.

- Line 326: remove one "the"

Corrected.

- *Line 330: units of dispersion in cm?*

In the context of the manuscript, the dispersion values refer to the R_{16} element of the beam transport matrix which maps the relative momentum deviation to a transverse spatial offset at the screen (i.e. $\Delta x = R_{16}\Delta p/p$). Since the relative momentum spread $\Delta p/p$ is dimensionless, the dispersion term has units of length

"At the closer screen, the horizontal dispersion (i.e. the linear transport matrix element which relates the horizontal offset at the screen with the relative momentum deviation) is 10 cm, while at the far screen we have an horizontal dispersion of 32.8 cm."

- *Line 345 vs 347: is n=0 "mode" or "peak"?*

Changed to mode

- *The caption of Fig.4 mentions "gamma_res" and "Delta E_res", but these are not defined in the text.*

We have changed the figure/caption to refer to 90% of the maximum energy, instead of 'gamma_res'. We have altered the caption to omit 'delta E_res', instead referring to the 'resonant energy gain', as labeled in the figure.

'Simulation results showing the proportion of electrons which reach 90\% of the resonant energy gain as a function of the resonant energy gain, colored according to $\Delta\phi$, the actual tunable parameter in this scheme.'

- *Also in Fig.4, there are curves for "Gain" and "Loss" but I am unclear as to the exact definition of these. Are these measured from the nominal (injection) energy to the highest/lowest measured electron energy? Please define them.*

We added a sentence: "Here, gain refers to the maximal observed energy gain relative to the nominal energy, and loss to the maximal observed loss. "

- *In your discussion, lines 296-297, please note the nominal (injection) energy used for these simulations here.*

We specify that the input beam energy used in the simulations is 6.1 MeV

Reviewer #3:

This manuscript reports on dynamic control of relativistic electron acceleration in double-grating structures. The authors employ spatial light modulators (SLM) to tailor incident light phases and amplitudes, thereby enabling phase-matching, pondermotive focusing, and alternating phase focusing (APF). This approach differs from conventional methods that rely on photonic nanostructures fabrication, offering a potentially more flexible and convenient way to manipulate both the transverse and longitudinal dynamics of electron acceleration.
The concept is interesting and could represent an important step toward versatile control of electron acceleration in DLA. However, based on the data currently presented, the advantages of beam control using SLM are not sufficiently clear. Several key issues should be addressed to strengthen the manuscript.

1. The authors claim 0.55 MeV energy gain, but this energy gain is not observed by using SLM. What's the laser damage threshold of SLM?

The damage threshold is 138 mJ/cm^2, but this is not the origin of the lower energy gain. Importantly, the measured reflectivity of the SLM is only 55%, the fill factor 93%, and the nominal diffraction efficiency with a linear ramped phase of 7.5 mrad is 90%. Additionally, the fresnel lens is not perfectly efficient at focusing all of the light into the narrow x-spot, and the optical axis of the SLM required the insertion of two additional half wave plates, each of which further reduced power throughput. This means that the overall intensity dropped by more than 60% by inserting the SLM, which is reflected in our inability to increase our maximal energy gain despite greatly increasing the interaction length.

This information is reported in the Spatial Light Modulator subsection of Methods.

2. In fig.3, only maximum energy gains are present. One can conclude less energy-gain with the sinusoidal modulation on SLM. However, to demonstrate the pondermotive focusing, more data should be provided, such as the full electron energy spectra, the images of transverse electron distributions, the changes of output electron current.
3. Similarly, in the demonstration of APF, only maximum energy gains are presents. Could authors provide more experimental data to support APF?

See comment 6 to referee # 1. We fully concur with the reviewers that demonstrating increased transmission would be the ideal direct evidence for improved confinement. Unfortunately, in the current experimental setup, evidence for focusing is strongly suppressed by the temporal overlap factor, and ultimately completely obscured by the large instability in transmitted beam charge.

Specifically, the temporal overlap between the electron bunch (~ 1 ps) and the laser pulse (~ 100 fs) means that at most 10% of the electron charge interacts with the focusing field.

Simulations for the parameters used in the experiment predict at best a transmission enhancement of <10% for the interacting slice.

However, experimentally, we observe shot-to-shot transmission fluctuations of approximately ±12\%, driven by incoming charge stability (2.4 ±0.2 pC as measured by an upstream toroid) coupled to pointing and energy jitter which decorrelate the transmission from the input charge (linear fit R^2 ~0.41). Consequently, the predicted small focusing effect on a thin slice of the beam is entirely buried within the baseline noise of the unmodulated majority of the beam.

While we could not directly measure the integrated current increase, the angular scan data for the ponderomotive sinusoidal phase modulation (Fig. 3) confirm that many non resonant modes were successfully excited. Since the longitudinal and transverse forces are coupled, the observation of the characteristic energy modulation signatures provides indirect but physically robust evidence that the corresponding focusing forces were present, even if the integrated transmission signal was below the detection threshold.

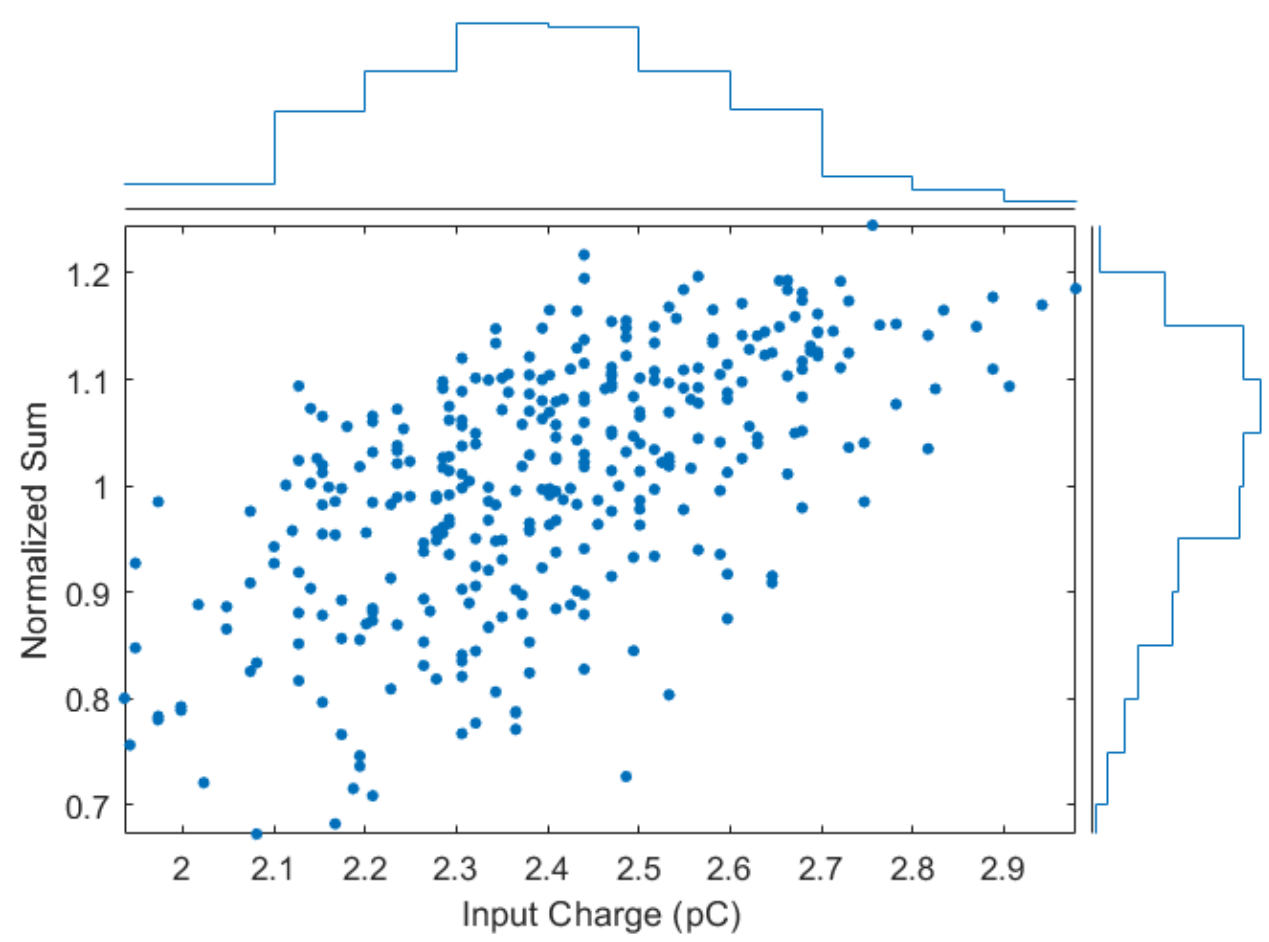

Figure 1. Transmitted beam intensity (measured as the sum of pixel counts from the spectrometer camera vs. input beam charge. The small correlation is not sufficient to eliminate the noise in the transmission data.

The paper has been amended to explain better this point
“In the experiment, due to pointing jitter and the mismatched phase space of the incoming e-beam, particle throughput was highly variable; consequently, we were not able to distinguish the relatively small expected increase in integrated current from the larger shot-to-shot charge fluctuations of the portion of the electron beam not temporally overlapping with the laser. Nevertheless, we observed clear signatures of both of these focusing schemes in the energy spectra of the beams, providing insights into the underlying beam dynamics.”

More detailed comments below:

4.The electron microscopy image of DLA structure is missing.

These are essentially commercial diffraction gratings which have been made to order such that the periodicity matches the 780nm laser. In Methods:Structure, we reference ‘All Optical Characterization of a Dual Grating Accelerator Structure’ by Crisp. et al, which goes more into detail on the assembly process. Since the gratings are standard and made by a commercial vendor, we did not do additional measurement after procurement. We have added a phrase ‘manufactured by Ibsen Photonics’ in the methods section to clarify that these gratings are commercially available.

5. Fig.2a presents the incident-angle dependence of the maximum electron energy gain. However, the use of polylines in the plots obscures the underlying experimental data, making it difficult to evaluate the results directly. It would be helpful if the authors could show the raw data points more clearly. In addition, an explanation why the maximum energy gain is dependent on z location is missing.

We have added dots to more clearly distinguish each data point. In addition, we have added a sentence to clarify the energy dependence on z position. ‘’The energy gain is non-uniform along z due to the variation of the optical intensity along the long laser axis.”

6. The z location of fig.2c is ~3.4mm, however, the z location of corresponding laser profiles in fig.2b is 4.25mm, since it corresponds to the second lineout in fig.2a.

This has been corrected.

7. In fig. 3b, the simulations show the electrons gain the same energy at $\Delta\theta=0$. However, the interaction length varies from 0.5mm to 5mm. Does the intensity of light field is also changed?

Yes, in order to highlight the effect of the change in width of the curve we normalize the intensity of the laser to produce the same maximal energy gain. We have added a sentence ‘The laser intensity is normalized for each interaction length to produce the same maximal energy gain at $\Delta\theta=0$.’ to clarify.

8. In fig. 3d, the meaning of the dashed line is not explained. The authors should clarify what this line represents in the figure.

There is no dashed line in 3d. We added clarifications in the text about the dashed lines in 3b and 2d.

For 2d: “ Maximum energy gain as a function of illumination length, comparing the curved, tuned phase profile indicated in solid lineouts to a linear tilt, indicated by the dashed lineouts.”

For 3b: “The dashed lines indicated interaction lengths which do not match the observed energy modulation peak width.”

9. In the line 223, how to obtain the 'effective interaction length 2.5±0.5mm' from those |sinΔθ | function-liked curves?

We thank the reviewer for this question. The estimation of the interaction length is based on the fundamental relationship between the longitudinal interaction window and the acceptance bandwidth of the accelerator. Theoretically, the energy gain ΔE as a function of the phase mismatch which in our case is linearly dependent on the angular detuning $\Delta\theta$ is proportional to the Fourier transform of the longitudinal field profile. For a uniform interaction over a length L, this results in a sinc function profile i.e. $\Delta E \propto \mathrm{sinc}\,(k\Delta\theta\, L/2)$.

Consequently, the width of the energy gain peak in the angular scan is inversely proportional to the interaction length L and a longer interaction results in a narrower angular acceptance. To estimate the interaction length, we modeled the expected energy gain vs angle relation for interaction lengths between 0.5 mm to 5 mm and assessed the root mean square error between the modeled energy gains and the data, excluding the central peak for which the energy gain is beyond the screen. As in 3d, we normalized the electric field by the interaction length to keep the maximal energy gain constant. For every scan, the minimum RMSE was obtained for either a 2.5 mm or 3.0 mm interaction, with a clear decrease in RMSE for modeled interaction lengths greater than 2 mm.

The error bar accounts for the uncertainty in the fit and the finite sampling of the angular scan.

The text has been reworded to clarify this point

"When no phase modulation is added, the scan reveals a single peak at $\Delta\theta$ = 0 (Fig. 3b). The dependence of the energy gain on the angular detuning $\Delta\theta$ follows a sinc-like function, the width of which is inversely proportional to the interaction length. The same is true for the peak widths in the ponderomotive excited case. By minimizing the root mean square error between a ballistic interaction model, normalized for equal energy modulation, and the data, we estimate an effective interaction length of 2.5± 0.5 mm."

10. The light intensity directly affects the electron transverse focusing length and drift length. In fig. 4d, I_0 is arbitrary units, what is the absolute value of light field?

The incident light field on the structure for this case was 1.8 GV/m corresponding to a maximal acceleration gradient of 88 MeV/m

11. It is not clear whether the use of a pulse-front-tilted laser has any influence on the spatial modulation introduced by SLM. The authors should clarify if and how pulse-front tilt affects the effectiveness or accuracy of the phase and amplitude control.

The pulse front tilt is essentially required to make any use of the functionality of the SLM. In absence of pulse front tilted laser, the interaction length would be limited to only the temporal length of the laser pulse, which at 100 fs is only $\sqrt{2}c\tau = 42$ microns. In terms of whether the

spatial phase modulation is affected by the pulse front tilt, that strongly depends on the optical system utilized. In our setup, but in the design phase and in the experimental implementation, we take particular care of imaging the PFT produced at the grating plane onto the SLM and then relay-image this plane to the interaction region in order to avoid any impact at first order in the linear transport. Chromatic aberrations, misalignments and other higher order effects in the transport can lead to a loss of fidelity in the control of the amplitude and phase of the field experienced by the electrons, but these problems are not specific to the PFT configuration, but common to any SLM-based optical system for large bandwidth laser pulses.
